# Intraoperative Optical Coherence Tomography-Guided Bleb-sparing Epithelial Exchange: A Modified Approach

**DOI:** 10.18502/jovr.v16i3.9447

**Published:** 2021-07-29

**Authors:** Dewang Angmo, Jyoti Shakrawal, Ramanjit Sihota

**Affiliations:** ^1^Glaucoma Research Facility & Clinical Service, Dr Rajendra Prasad Centre for Ophthalmic Sciences, All India Institute of Medical Sciences, New Delhi, India

**Keywords:** Bleb-sparing Epithelial Exchange, iOCT, Thin Avascular Blebs

## Abstract

With the advent of newer technologies, real-time anterior segment optical coherence tomography (OCT) integrated with the operating microscope has become possible. We are proposing the technique of bleb revision with greater precision under direct visualization of bleb anatomy and extent of tissue depth allowing better localization and easy maneuvering with lesser complications. In this surgical technique, bleb revision was performed using intraoperative real-time OCT incorporated in OPMI LUMERA 700 microscope. Live surgical and OCT view were seen on a common screen together. A moderately elevated, diffuse functional bleb was noted after three months of bleb revision in both cases with controlled intraocular pressure. Intraoperative OCT-guided bleb-sparing epithelial exchange is an adjunctive technique for bleb repair surgery with an increased precision of surgery which can reduce complications, minimize surgical time and maximize surgical outcome.

##  INTRODUCTION

Long-term results of trabeculectomy have improved over time with modifications to surgical technique,^[1]^ application of antifibroblastic agents like mitomycin-C (MMC), and use of releasable sutures. The intraoperative use of MMC and 5-fluorouracil in trabeculectomy has increased the susceptibility of thin avascular blebs.^[2]^ These thin avascular blebs are more prone to vision-threatening complications such as hypotonic maculopathy, blebitis, and endophthalmitis, especially in pediatric age groups. Ehlers et al stated that the use of microscope-mounted intraoperative optical coherence tomography (iOCT) can modify the surgical decisions of both anterior and posterior segment surgeries.^[3,4]^ Various surgical or nonincisional methods using cyanoacrylate glue or autologous blood injections for bleb revision have been described.^[5,6,7]^ Our technique of bleb-sparing epithelial exchange (BSEX) is well established and safe.^[8,9]^

The use of iOCT is a noninvasive tool evolving for different glaucoma surgical interventions like trabeculectomy, goniosynechialysis, Ahmed glaucoma valve (AGV) implantation, bleb needling, etc. In this technique, epithelial peeling of a thin avascular bleb followed by conjunctival advancement is done, which preserves the functional bleb without excising it and thereby less need of glaucoma medications postoperatively.^[8]^ However, the trypan blue dye used for staining the epithelium causes difficulty in visualizing the epithelium and thereby dissection. This may either lead to incomplete epithelium removal or damage to the underlying bleb structure. Peeling in multiple fragments is required in these cases to avoid inadvertent damage to the underlying bleb structure, which might not ensure complete removal.^[8]^ Therefore, we used iOCT in adjunct to trypan blue dye to ensure complete epithelial peeling without causing any inadvertent damage to the underlying functional bleb structure.

##  SURGICAL METHOD

Bleb revision was done after taking a written informed consent, using an iOCT RESCAN 700 (Carl Zeiss Meditec, Germany) fused with OPMI LUMERA 700 microscope (Carl Zeiss Meditec, Germany). The Calisto eye displays the live surgical and OCT view on the common screen together.

**Figure 1:**
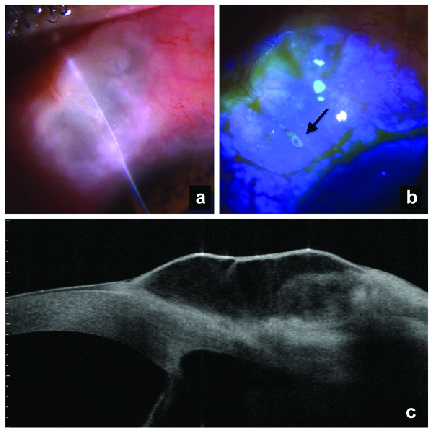
Clinical photograph of the bleb showing (a) a very thin, avascular and, multicystic bleb. (b) fluorescein staining of the bleb showing a pinpoint leak (black arrow). (c) ASOCT image of the bleb.

**Case 1:** A 58-year-old male with primary open-angle glaucoma presented with a past history of right eye (RE) trabeculectomy seven years back. On examination, RE vision was 6/9 on Snellen's visual acuity chart and IOP was 8 mmHg. Slit lamp examination showed a thin avascular bleb with sweating on fluorescein staining. [Figures 1a and 1b]. Bleb anterior segment optical coherence tomography (ASOCT) showed an overhanging and elevated bleb [Figure 1c]. RE iOCT-guided bleb revision was performed.

**Figure 2:**
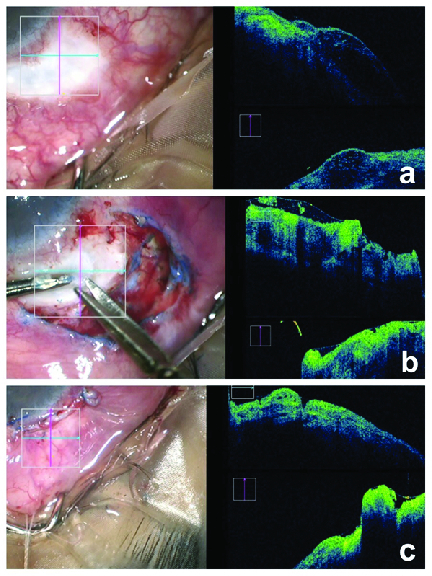
(a) Preoperative clinical photograph of the avascular bleb with the corresponding live iOCT imaging-horizontal scan (blue horizontal line) and vertical scan (pink vertical line) of the bleb area showing large bleb height, a thin epithelium, and multiple hyporeﬂective spaces in the bleb. (b) Intraoperative clinical photograph showing the lightly stained bleb epithelium with trypan blue dye, being peeled off. The corresponding live iOCT imaging of the bleb area shows an easily visible thin hyper-reflective epithelium. (c) Postoperative clinical photograph showing a bleb covered by adjacent healthy conjunctiva. The corresponding live iOCT imaging of the bleb area shows reduced bleb height, a thick epithelium, and few hyporeﬂective spaces within the bleb tissue.

**Figure 3:**
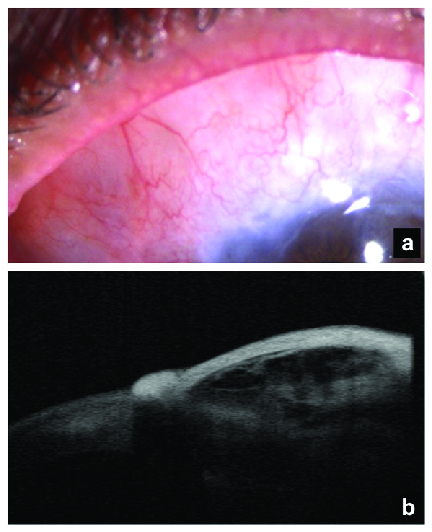
(a) Postoperative clinical photograph showing a moderately elevated and vascular, diffuse functional bleb after three months. (b) Postoperative ASOCT image showing decrease in the bleb height, a thicker epithelium, and less hyporeﬂective spaces within the bleb tissue.

**Case 2:** A 36-year-old female with juvenile open-angle glaucoma presented with left eye (LE) IOP of 4 mmHg. She had a history of both eyes trabeculectomy done one year ago. On slit lamp examination, a thin avascular bleb with a bleb leak was present. She also complaint of drop in vision of LE and her visual acuity on presentation was 1/60, with evidence of hypotonic maculopathy visible in fundus. She underwent LE iOCT-guided bleb revision.

### Surgical Technique

After anesthetizing the eye with peribulbar block (0.5% bupivacaine + 2% lignocaine), a wire speculum was inserted. A curved Vanna's scissor was used to cut the conjunctiva along the lateral and the posterior margins of the avascular bleb. Hemostasis was achieved and the surrounding normal conjunctiva was separated from the underlying Tenon's with a blunt-tipped Westcott's scissor, until its free margin could be advanced easily to the limbus. Care was taken to avoid buttonholing of the conjunctiva. Trypan blue dye (0.06%) was applied to stain the epithelium over the bleb. The anterior edge of the bleb epithelium was identiﬁed, and only the stained epithelium was gradually peeled off the surface backward and horizontally under direct visualization of the tissue depth on iOCT. This prevented damage to the underlying functional bleb structure [Figure 2b]. As the raised epithelial sheet reached the more ﬁbrosed posterior margin of the bleb, it was cut off. Thereafter, the adjacent conjunctiva was advanced and hitched to the limbus with 6-0 vicryl horizontal mattress sutures. A watertight closure of the conjunctival ﬂap was achieved by continuous 8-0 vicryl sutures at the lateral edges avoiding dog ears and two 10-0 nylon horizontal mattress sutures, buried across the limbal extent. *In vivo*, preoperative and postoperative assessment of the bleb was done on iOCT [Figures 2a and 2c]. Postoperatively, topical antibiotic–steroid combination eyedrops and ointment were prescribed for four weeks. Both patients were reviewed at day 1, week 1, month 1, and month 3 after the surgery.

##  RESULTS

The first case after bleb revision showed an RE diffuse and vascular bleb with an IOP of 14 mmHg on postoperative day 1. RE vision was maintained at preoperative levels at three months follow-up. Bleb sweating was absent. A moderately elevated, diffuse functional bleb was noted after three months of bleb revision with RE IOP of 12 mmHg. The ASOCT of the ﬁltering blebs postoperatively showed a decrease in the bleb height, a thicker epithelium, and smaller hyporeﬂective spaces within the bleb tissue [Figures 3a and 3b]. The second patient also showed a functional bleb at four months postoperatively. The vision in LE improved to 6/24 with resolution of hypotonic maculopathy.

##  DISCUSSION

Introduction of OCT into clinical practice brought a landmark change in diagnosis, management, and monitoring of nearly all ocular pathologies. Along with that, OCT integrated with operating microscope resulted in a paradigm shift in ocular surgery. The feasibility of iOCT was first described by Ehlers et al.^[4]^ Ever since, its use in ophthalmology has evolved. iOCT has been used for bleb needling for an encysted bleb with high IOP after trabeculectomy, important guide during crucial steps of lamellar keratoplasty, as well as during stromal dissection in ocular surface squamous neoplasia.^[10,11]^

During surgical maneuvering, the iOCT with heads up display (HUD) allows rapid visualization of the area of interest and provides the surgeon with information regarding instrument and tissue interactions. Because of its finer resolution, OCT is able to present detailed view of the very thin bleb wall, and an accurate assessment of separating epithelium from functioning bleb structure.

The epithelium can be precisely identified and dissected under direct visualization without causing any inadvertent damage to the underlying bleb structure. This permits better maneuvering, improved dissection of the anterior and posterior bleb wall under visualization, thereby reducing the risk of complications. Moreover, iOCT may also be useful in visualizing any underlying scleral defect. However, repeated microscope adjustments for a better focus of the area of interest and obscuration of the operation site due to conjunctival bleeding may cause difficulties during the procedure.

iOCT ensures complete epithelial peeling of the deceased epithelium, which is important to prevent postoperative increase in IOP.^[8,12]^ It also reduces the direct trauma to the underlying bleb structure and can also be used as an excellent teaching tool for trainees. However, the high cost and availability of iOCT are the major limitations. This technique could be applied to a wider range of patients including all age groups.^[13]^ In summary, iOCT-guided bleb-sparing epithelial exchange is an adjunctive technique for bleb repair surgery which can maximize the surgical outcome, minimize surgical time, and reduce complications with an increased precision of surgery. Further studies with a larger sample size and a longer follow-up along with a comparison to standard procedures are recommended.

##  Financial Support and Sponsorship

Nil.

##  Conflicts of Interest

There are no conflicts of interest.

## References

[B1] Khaw PT, Chiang M, Shah P, Sii F, Lockwood A, Khalili A (2012). Enhanced trabeculectomy – the Moorfields Safer Surgery System. Dev Ophthalmol.

[B2] Singh K, Mehta K, Shaikh NM, Tsai JC, Moster MR, Budenz DL, et al (2000). Trabeculectomy with intraoperative mitomycin C versus 5-fluorouracil. Prospective randomized clinical trial Ophthalmology.

[B3] Kumar RS, Jariwala MU, Venugopal JP, Puttaiah NK, Balu R, Shetty R, et al (2015). A pilot study on feasibility and effectiveness of intraoperative spectral-domain optical coherence tomography in glaucoma procedures. Transl Vis Sci Technol.

[B4] Ehlers JP, Dupps WJ, Kaiser PK, Goshe J, Singh RP, Petkovsek D, et al (2014). The prospective intraoperative and perioperative ophthalmic imaging with optical coherence tomography (PIONEER) Study: 2-year results. Am J Ophthalmol.

[B5] Pazos M, Dyrda A, AntÃ³n A (2015). Bleb revision for resolution of hypotony maculopathy following primary trabeculectomy. Am J Ophthalmol.

[B6] Lin AP, Chung JE, Zhang KS, Chang MM, Orengo-Nania S, Gross RL, et al (2013). Outcomes of surgical bleb revision for late-onset bleb leaks after trabeculectomy. J Glaucoma.

[B7] Burnstein AL, WuDunn D, Knotts SL, Catoira Y, Cantor LB (2002). Conjunctival advancement versus nonincisional treatment for late-onset glaucoma filtering bleb leaks. Ophthalmology.

[B8] Sihota R, Angmo D, Sen S, Gupta V, Dada T, Pandey RM (2016). The long-term outcome of primary “Bleb-sparing, Epithelial Exchange” in dysfunctional filtering blebs. J Glaucoma.

[B9] Dada T, Midha N, Shah P, Sidhu T, Angmo D, Sihota R (2017). Innovations in glaucoma surgery from Dr. Rajendra prasad centre for ophthalmic sciences Indian J Ophthalmol.

[B10] Dada T, Angmo D, Midha N, Sidhu T (2016). Intraoperative optical coherence tomography guided bleb needling. J Ophthalmic Vis Res.

[B11] Titiyal JS, Kaur M, Falera R (2017). Intraoperative optical coherence tomography in anterior segment surgeries. Indian J Ophthalmol.

[B12] Sihota R, Dada T, Gupta SD, Sharma S, Arora R, Agarwal HC (2000). Conjunctival dysfunction and MMC induced hypotony. J Glaucoma.

[B13] Sihota R, Selvan H, Sidhu T, Kamble N, Angmo D, Yadav S, et al (2020). Clinical and ASOCT evaluations of ’bleb-sparing epithelial exchange’ in paediatric and adult dysfunctional blebs over 5 years. Graefes Arch Clin Exp Ophthalmol.

